# The need for COVID-19 clinical trials in LMICs

**DOI:** 10.3389/fpubh.2022.1038840

**Published:** 2023-01-09

**Authors:** Fatima Saleh

**Affiliations:** Department of Medical Laboratory Sciences, Faculty of Health Sciences, Beirut Arab University, Beirut, Lebanon

**Keywords:** COVID-19, dexamethasone, clinical trials, Lebanon, low- and middle-income countries (LMIC)

Since the outbreak of the COVID-19 pandemic, the disease has spread worldwide with more than 603 million confirmed cases and with a death toll surpassing 648 million as of 6 September, 2022.^1^ Now in its third year, the pandemic is far from finished as the virus continues to claim victims and seize lives. Despite vaccine development and success, potential emergence of SARS-CoV-2 vaccine-resistant variants as well as waning of vaccine-induced immunity, may compromise the impact of vaccines and thus necessitate the need for therapeutics that can control the disease and save lives alongside vaccines.

Early in the pandemic, dexamethasone was proposed as a potential treatment for patients with severe and critical COVID-19 as it suppresses the “cytokine storm” induced by SARS-CoV-2. Given the large number of clinical studies supporting the use of dexamethasone in COVID-19 patients, the most robust evidence came from the Randomized Evaluation of COVID-19 Therapy (RECOVERY) trial conducted by researchers at Oxford University. Low dose dexamethasone (6 mg once daily) has been proven to reduce the 28-day mortality rate for COVID-19 patients on either invasive mechanical ventilation or oxygen therapy (1). On April 8, 2021, the UK National Institute for Health and Care Excellence (NICE) recommended oral or intravenous administration of low dose of dexamethasone to severe or critically ill COVID-19 patients needing supplementary oxygen.^2^ Similarly, dexamethasone use has also been recommended by the Infectious Diseases Society of America (IDSA) (2), the US National Institutes of Health (NIH)^3^ and the WHO^4^ for hospitalized patients with COVID-19 requiring oxygen therapy. It is worth noting that these therapeutic recommendations were based on evidence acquired from clinical trials conducted in high-income countries and subsequently extrapolated to treatment of COVID-19 patients in low- and middle-income countries (LMIC) (1, 3). Despite the large number of trials for effective treatment for COVID-19, only a very small portion has been conducted in LMICs (4), which is very essential as outcomes from patients in LMICs could differ due to genetic variations among SARS-CoV-2 strains and other host and healthcare system factors specific to the LMIC settings. The inter-individual variability in dexamethasone response has been reported at the genomic and transcriptomic levels. At the DNA level, SNPs in genes such as NR3C1, NR3C2, and ABCB1 have been linked to an altered dexamethasone metabolism (5). Moreover, dexamethasone induces significant changes in the transcriptome of treated individuals. Therefore, there is a need to conduct dexamethasone clinical trials on different populations, especially those residing in LMICs.

For COVID-19 patients in LMICs such as Lebanon, cost and availability of treatment is very essential. Lebanon is a country facing unprecedented economic crisis that is pushing the country's healthcare system to the brink and creating disastrous medicine shortages. Therefore, for Lebanon, having an effective treatment such as dexamethasone, which is at the same time inexpensive, is highly significant.

## Author contributions

The author confirms being the sole contributor of this work and has approved it for publication.

## Footnotes

^1^*WHO Coronavirus Disease (COVID-19) Dashboard*. Available online at: https://covid19.who.int/ (accessed November 12, 2020).

^2^*Overview | COVID-19 Rapid Guideline: Managing COVID-19*. NICE Guidance. Available online at: https://www.nice.org.uk/guidance/ng191 (accessed September 7, 2022).

^3^*COVID-19 Treatment Guidelines 2*. Available online at: https://www.covid19treatmentguidelines.nih.gov/ (accessed May 28, 2021).

^4^*Corticosteroids for COVID-19*. Available online at: Available online at: https://www.who.int/publications/i/item/WHO-2019-nCoV-Corticosteroids-2020.1 (accessed September 7, 2022).
